# Specific overexpression of contactin-associated protein-like 2 and its effects on pain-related behaviour in mice

**DOI:** 10.1097/PR9.0000000000001309

**Published:** 2025-06-26

**Authors:** Mandy Tseng, Steven J. Middleton, Adham Farah, Sarosh R. Irani, John M. Dawes, David L. Bennett

**Affiliations:** a Nuffield Department of Clinical Neurosciences, University of Oxford, Oxford, United Kingdom; Departments of b Neurosciences and; c Neurology, Mayo Clinic, Jacksonville, FL, USA

**Keywords:** CASPR2, Neuropathic pain, Kv1 channels, Capsaicin, Pain

## Abstract

**Introduction:**

Hyperexcitability, particularly of DRG neurons, is a key driver of persistent pain states including neuropathic pain. Contactin-associated protein-like 2 (CASPR2) is transmembrane protein known to interact and regulate the function of Kv1 channels, important determinants of neuronal excitability. Patients with autoantibodies (-Abs) against CASPR2 commonly have neuropathic pain, and these patient-Abs enhance the excitability of DRG neurons secondary to Kv1 channel disruption, leading to pain-related hypersensitivity. This is also observed after genetic ablation of CASPR2. Conversely, increasing CASPR2 levels in DRG neurons reduces excitability.

**Objectives:**

The aim of this study was to assess whether overexpressing CASPR2 could be a potential approach to treat pain.

**Methods:**

We generated a Cre-dependent mouse line to express human CASPR2 (R26^LSL:hCNTNAP2(+/+)^ (CASPR2^OE^)) and crossed this with either Hoxb8^Cre^ or Nav1.8^Cre^ mice for CASPR2 overexpression in either all DRG neurons or to target nociceptors more selectively.

**Results:**

In both lines, CASPR2 was significantly overexpressed in DRG neurons, including at the cell membrane. In comparison to control littermates, overexpression of CASPR2 did not affect acute pain–related behaviour to mechanical or thermal stimulation or nerve injury–induced pain-related hypersensitivity. However, in both overexpression mouse lines, there were significantly reduced nocifensive responses to capsaicin. Although CASPR2 overexpression increased the contribution of α-dendrotoxin–sensitive Kv1 channels to the slowly inactivating outward current I_KD_, it did not affect total I_KD._

**Conclusion:**

CASPR2 overexpression in DRG neurons can affect nociception, reducing pain-related behaviours to the noxious stimulant capsaicin, yet is insufficient to reduce mechanical pain–related hypersensitivity caused by nerve injury.

## Abstract 2

Supplemental Digital Content is Available in the Text.

Using mouse models, the conditional overexpression of contactin-associated protein-like 2 in DRG neurons does not affect neuropathic pain behaviours but reduces sensitivity to the noxious stimulant capsaicin.

## 1. Introduction

Neuropathic pain is a common chronic condition that affects 7% to 10% of the general population,^4,20^ with an urgent need to test and develop novel targeted therapies for more efficacious treatment. Hyperexcitability in DRG neurons is a significant contributor to neuropathic pain conditions,^18^ mediated by alterations in ion channel function and expression. Voltage-gated potassium channels (VGKCs), in particular Kv1 channels (eg, Kv1.1 and 1.2), are essential in limiting neuronal excitability and are known to play an important role in determining DRG neuron activity.^37^ Accordingly, genetic disruption or pharmacological blockade of Kv1 channels leads to pain-related hypersensitivity.^8,17^ These channels are considered important in neuropathic pain. Kv1.1 and 1.2 are downregulated in the DRG by peripheral nerve injury coincident with neuronal hyperexcitability and have been shown to play a key role in the development of neuropathic pain in preclinical models.^1,24,30,40,45^ Meanwhile, Kv1.6 expression is suggested to contribute to neuropathic pain resolution.^5^ Contactin-associated protein-like 2 (CASPR2) is a type I transmembrane protein and part of the VGKC complex. Its interaction with Kv1 channels is well established, including the subunits Kv1.1, 1.2, and 1.6.^7^ This interaction involves its partner protein contactin-2, and CASPR2 is vital for the correct localisation and clustering of Kv1 channels in peripheral nerves.^21,28^ A significant proportion of patients with autoantibodies (-Abs) against CASPR2 develop neuropathic pain, which can be relieved with immunotherapy.^14,16,22,23,29,34^ This suggests a causal role of CASPR2-Abs in the development of pain. Indeed, the passive transfer of patient CASPR2-Abs in mice recapitulates neuropathic pain-like behaviour.^10^ This effect is not associated with inflammation or nerve damage, but instead increased excitability due to Kv1 channel disruption.^10^ Furthermore, multiple studies have shown that genetic ablation of CASPR2 in mice causes increased excitability of DRG neurons and pain-related hypersensitivity.^10,41^ This increased excitability after CASPR2 ablation is correlated with reduced Kv1 currents, due to a loss of Kv1 channel expression at the cell membrane, suggesting CASPR2 as an important mediator in the trafficking of these channels.^10^ Given their importance in the pathogenesis of neuropathic pain, modulation of Kv1 channels may remedy pain after nerve injury. For example, both preventing the downregulation of Kv1.2 and inducing its overexpression can attenuate pain-related hypersensitivity after nerve injury in mice.^13,45^ The dissociation and culture of DRG neurons leads to the downregulation of CASPR2 and subsequent hyperexcitability, which can be reversed with CASPR2 overexpression via the rescue of Kv1 currents.^10^ Considering these observations and the influence of CASPR2 on multiple Kv1 channel subunits, we wanted to assess the impact of CASPR2 overexpression on pain-related behaviour in mice using transgenic models for conditional overexpression in DRG neurons.

## 2. Methods

### 2.1. Animal care and study design

All procedures were conducted in accordance with UK home office regulations and the Animals Scientific Procedures Act 1986 at a licensed facility within the University of Oxford. Our animal studies are reported in accordance with the ARRIVE guidelines. Animals were group housed in individually ventilated cages on a 12-hour light–dark cycle in temperature and humidity controlled rooms, with food and water available ad libitum. Both male and female mice were used in equal numbers. No animals were excluded. Before behavioural tests, mice were acclimatised to their testing environment and equipment for a minimum of 30 minutes. Mice were assigned to experimental groups based on genotype, and littermates were used as controls for all studies. For behavioural testing, mice were tested at a consistent time of day in the same designated room. Mice were randomly assigned to testing boxes each day and tested in a random order. The experimenter was blind to animal genotype before testing and until after behavioural analysis was complete. Samples sizes were chosen based on a power calculation using historical lab data relating to mechanical and thermal threshold responses, an α error of 0.05, a power of >80%, and the assumption that an effect size of 25% would be biologically meaningful. For culture experiments, mice were used between the ages of 6 and 10 weeks. Behaviour experiments were conducted on mice between 3 and 7 months of age.

### 2.2. Mouse lines

All mice were maintained on a C57Bl/6J background. The transgenic mouse line R26^LSL:hCNTNAP2(+/+)^ (CASPR2^OE^) was generated by Dr Ben Davies (Wellcome Trust Centre for Human Genetics, Oxford, United Kingdom). It contains a floxed stop codon upstream of an open reading frame for exogenous human (hu) CASPR2 placed in the Rosa26 locus under the control of the neuronal CAG promoter. The Cre-dependent removal of the stop codon will drive the overexpression of huCASPR2 in specific neuron populations expressing the Cre recombinase enzyme.

The CASPR2^OE^ line was crossed with a Hoxb8^Cre^ line^39^ to generate Hoxb8^Cre^:CASPR2^OE^ mice for the overexpression of CASPR2 in all DRG neurons (caudal to C4 spinal segment). Similarly, CASPR2^OE^ mice were crossed with Nav1.8^Cre^ line^35^ to overexpress CASPR2 more selectively in nociceptors (Na_v_1.8^Cre^:CASPR2^OE^). Cre^+^:CASPR2^OE^ mice were bred with Cre^−^:CASPR2^OE^ mice to ensure that all offspring were experimentally relevant (ie, 50% Cre^+^, 50% Cre^−^). Cre recombinase in all transgenic mice was detected by PCR of genomic DNA. Primers for Cre (Forward 5′-agc​ctg​ttt​tgc​acg​ttc​acc-3′, Reverse 5′-ggt​ttc​ccg​cag​aac​ctg​aa-3′) and PCR control primers (Forward 5′-cct​agc​acc​cac​cca​aag​agc​tg-3′, Reverse 5′-ggt​cct​cac​tgg​cag​cag​ctg​ca-3′).^25^

### 2.3. Behavioural testing

#### 2.3.1. Von Frey hairs

Mechanical sensitivity was assayed using Von Frey filaments. Mice were randomly assigned and acclimatized to a test box (5 × 5 × 10 cm) elevated on a wire mesh base. Mice were tested on their plantar hind paws using calibrated filaments (Linton Instrumentation) and the “up-down” method^6,12^ to evaluate their 50% paw withdrawal thresholds.

#### 2.3.2. Dynamic brush

Mice were randomly assigned and acclimatized to a test box (5 × 5 × 10 cm) elevated on a wire mesh base. A small paintbrush (5-0, the Art Shop) was brushed on the plantar surface of the mouse hind paw from the heel to the toes at approximately 2 cm/second. Responses are scored on a scale from 0 to 3. 0: No response or moving or lifting of the paw for less than 1 second. 1: Sustained lifting of the paw or a single flinch. 2: Quick withdrawal and latera paw lift above the level of the body. 3: Intense, quick withdrawal of the paw accompanied by flinching and licking or biting of the paw.

#### 2.3.3. Hargreaves test

Mice were randomly assigned a test box (5 × 5 × 10 cm) elevated on glass floor and acclimatised. Tests were performed on the plantar surface of their hind paws using the Basile Plantar test apparatus (Ugo Basile, Italy), which provides a radiant laser heat source. The latency to paw withdrawal was recorded and taken as a measure of nociceptive threshold to radiant heat.

#### 2.3.4. 50/53°C hotplate

Mice were placed onto a Perspex enclosed Hotplate (Ugo Basile, Italy) set at 50 or 53°C and were observed until mice displayed pain-like behaviours on their hind paws (lifting, flicking, licking of the hind paw). The latency to respond was recorded with a cut-off of 20 seconds.

#### 2.3.5. Dry ice assay

Mice were placed into a randomly assigned test box elevated on a glass floor and acclimatized for 30 to 50 minutes. A 3-mL syringe was filled with dry ice. A cold stimulus was delivered by applying a dry iced-filled syringe to the glass underneath the paw and the latency to withdrawal was recorded.

For mechanical (Von Frey, dynamic brush), the Hargreaves test and the dry ice assay, averages were taken from 3 to 4 measurements per paw on 3 different days. For the hotplate test, 1 measurement was obtained per day on 3 different days.

#### 2.3.6. Capsaicin assay

Mice received a single intraplantar injection of 3 µg of capsaicin (Sigma-Aldrich, United States) diluted in sterile saline with 1% ethanol and 0.5% tween-20 (Sigma-Aldrich, United States) in a volume of 10 µL. Mice were placed in a Perspex cylinder and the duration of pain-related behaviour (biting, licking, flinching, or paw lifting) was recorded over a 5-minute period.

### 2.4. Whole cell patch clamp

Voltage clamp recordings were conducted at room temperature using an Axopatch 200B amplifier and Digidata 1550 acquisition system (Molecular Devices, United Kingdom). Data were low-pass filtered at 2 kHz and sampled at 10 kHz. Series resistance was compensated 75%–90% to reduce voltage errors. Filamental borosilicate glass (1.5 mm OD, 0.84 mm ID; World Precision Instruments, United Kingdom) patch pipettes were pulled (2–4 MΩ) and filled with internal solution (mM): 120 K^+^ gluconate, 20 KCl, 2 MgCl_2_, 10 EGTA, 10 HEPES, 1 CaCl_2_, and 5 MgATP; pH was adjusted to 7.3 with KOH, and osmolarity was set to 305 mOsm. Extracellular solution contained (mM): 150 choline-Cl, 5 KCl, 2 CaCl_2_, 1 MgCl_2_, 10 HEPES, 0.1 CdCl_2_, and 10 glucose; pH was adjusted to 7.4 with KOH, and osmolarity was set to 315 mOsm. α-Dendrotoxin (α–DTX, Alomone, Israel) was prepared in H_2_O, diluted in extracellular solution, and added via the perfusion system. Outward currents were elicited by depolarising the membrane potential from −70 to +40 mV for 500 milliseconds in 10 mV increments, after a 1-second prepulse conditioning step to −40 mV. The outward current generated at the end of the depolarising pulse was taken as I_KD_.

## 3. Results

### 3.1. Confirmation of contactin-associated protein-like 2 overexpression

**Figure 1.**
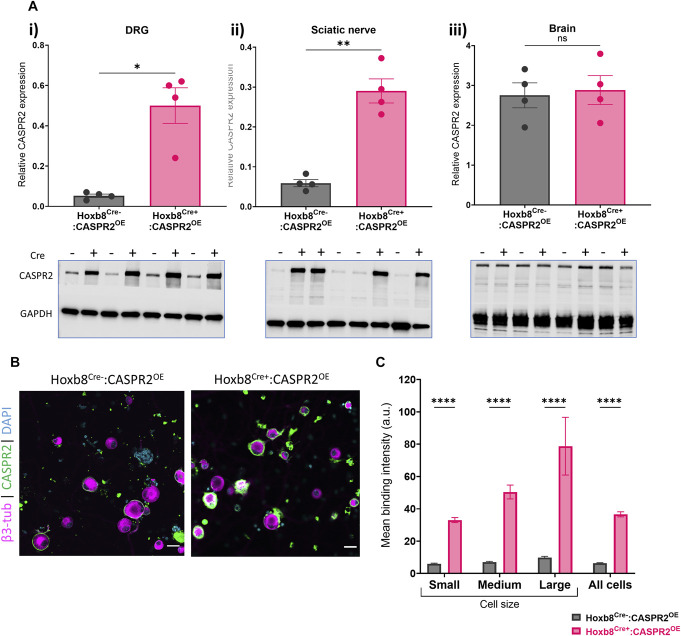
CASPR2 overexpression in Hoxb8^Cre+^:CASPR2^OE^ mice. (A) Quantification of CASPR2 protein in the i) DRG, ii) sciatic nerve, and iii) brain with immunoblots shown below (relative protein expression normalised to GAPDH). CASPR2 protein is overexpressed in the (A-i) DRG and (A-ii) sciatic nerve of Hoxb8^Cre+^:CASPR2^OE^ mice compared to Cre-littermate controls. (A-iii) CASPR2 is not overexpressed in the brain of Hoxb8^Cre+^:CASPR2^OE^ mice. Two-tailed unpaired *t* test, *P < 0.05, **P < 0.01, n = 4. Images of immunoblots shown below. (B) Representative images of CASPR2 expression (green) on the soma of Hoxb8^Cre−^:CASPR2^OE^ (left) and Hoxb8^Cre+^:CASPR2^OE^ (right) DRG neurons (cultured for 2 days in vitro). DAPI (cyan) and βIII-tubulin (magenta). Scale bar 25 μm. (C) Quantification of CASPR2 expression on the surface of DRG neurons, separated by cell size: small (<25 μm in diameter), medium (25–35 μm), and large (>35 μm). CASPR2 stained with an antibody targeting an extracellular epitope on live neurons. DRG neurons pooled from 2 animals/genotype CASPR2 is overexpressed on the surface of DRG neurons of all sizes from Hoxb8^Cre+^:CASPR2^OE^ mice (268 cells) compared to Cre^−^ control (135 cells). Two-way ANOVA with Šídák multiple comparisons test, ****P < 0.0001 vs Hoxb8^Cre−^:CASPR2^OE^ (n = cell). All data expressed as mean ± SEM. CASPR2, contactin-associated protein-like 2.

To evaluate the effect of CASPR2 overexpression, we generated the transgenic mouse line R26^LSL:hCNTNAP2(+/+)^ (hereafter referred to as CASPR2^OE^). This line has an exogenous human (hu) CASPR2 sequence placed in the Rosa26 locus of the mouse genome under the control of the neuronal CAG promoter to drive CASPR2 overexpression in specific neuron populations upon Cre-dependent removal of a floxed stop codon. These mice were crossed with a Hoxb8^Cre^ line (Hoxb8^Cre^:CASPR2^OE^) to specifically overexpress CASPR2 in all DRG and spinal neurons caudal to the C4 spinal segment.^39^ Immunoblots confirmed that CASPR2 was significantly overexpressed in the DRG (Fig. 1A-i) and sciatic nerve (Fig. 1A-ii) of Hoxb8^Cre+^:CASPR2^OE^ compared to littermate controls, without changes of CASPR2 expression in the brain (Fig. 1A-iii). To gain insights into whether this overexpression was functionally relevant, we next assessed the expression of surface CASPR2 on cultured DRG neurons with live-cell staining using an antibody, which binds the extracellular domain of CASPR2.^36^ Surface expression of CASPR2 was significantly higher in Hoxb8^Cre+^:CASPR2^OE^ DRG neurons compared to those from control (Hoxb8^Cre−^:CASPR2^OE^, Figs. 1B and C). The significant increase in expression was seen across all subtypes when divided by cell size (Figs. 1B and C), in line with expression of Hoxb8 in all DRG neurons.^39^ In parallel, we also generated a mouse line to more selectively target overexpression of CASPR2 to nociceptors (Nav1.8^Cre^:CASPR2^OE^).^35^ CASPR2 was significantly overexpressed in the sciatic nerve of Nav1.8^Cre+^:CASPR2^OE^ mice (Supplemental Figure S1A-i, http://links.lww.com/PR9/A324), with a trend towards overexpression in the DRG (Supplemental Figure S1A-ii, http://links.lww.com/PR9/A324). In vitro analysis confirmed the overexpression of surface CASPR2 on Nav1.8^Cre+^:CASPR2^OE^ DRG neurons compared to controls (Supplemental Figure S1B, C, http://links.lww.com/PR9/A324), largely restricted to small diameter DRG neurons (Supplemental Figure S1C, http://links.lww.com/PR9/A324). This verified that CASPR2 overexpression was targeted to nociceptors. These results validate the newly generated CASPR2^OE^ mouse line and confirm the significant and specific overexpression of CASPR2 in both Hoxb8^Cre+^:CASPR2^OE^ and Nav1.8^Cre+^:CASPR2^OE^ mice.

### 3.2. Contactin-associated protein-like 2 overexpression in the DRG does not alter acute pain–related behaviour to mechanical or thermal stimuli

**Figure 2.**
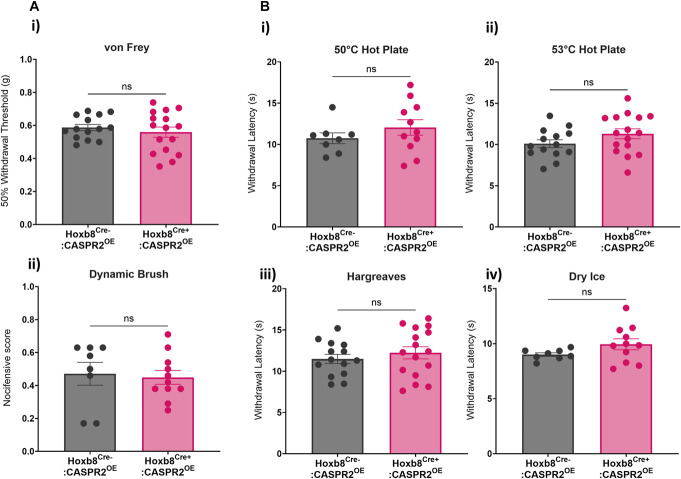
CASPR2 overexpression in Hoxb8^Cre+^:CASPR2^OE^ mice does not affect acute pain–related behavior. (A) There were no significant differences in mechanical sensitivity detected between Hoxb8^Cre+^:CASPR2^OE^ (n = 11–16) and Hoxb8^Cre−^:CASPR2^OE^ (n = 8–14) control littermates measured by either (A-i) von Frey hairs or (A-ii) dynamic brush. (B) There were no significant differences in thermal sensitivity between genotypes as measured by (B-i) 50°C or (B-ii) 53°C hot plate, (B-iii) Hargreaves test, or (B-iv) dry ice. Hoxb8^Cre+^:CASPR2^OE^, n = 11–16; Hoxb8^Cre−^:CASPR2^OE^, n = 8–14. Two-tailed unpaired *t* test. All data shown as mean ± SEM. CASPR2, contactin-associated protein-like 2.

To determine if overexpression of CASPR2 in DRG neurons affects mechanical or thermal sensitivities, behavioural testing was performed in adult Hoxb8^Cre^:CASPR2^OE^ mice. Pain-related mechanical sensitivity was assessed by measuring withdrawal thresholds to von Frey hair stimulation, with no differences observed between genotypes (Fig. 2A-i). There were also no differences in the responses to dynamic brush (Fig. 2A-ii), noxious heat (as measured by the hotplate and Hargreaves test [Fig. 2B-i-iii]), or noxious cold (Fig. 2B-iv). Given the broad expression of Hoxb8 in DRG neurons and spinal cord, we also assessed whether CASPR2 overexpression altered proprioception or motor activity: Hoxb8^Cre+^:CASPR2^OE^ mice displayed normal behaviour on the beam test, Rotarod and open field (Supplemental Figure 2A, http://links.lww.com/PR9/A324). For acute pain behaviours, similar findings were also observed in mice selectively overexpressing CASPR2 in nociceptors. No significant differences were observed for Nav1.8^Cre+^:CASPR2^OE^ mice when compared to littermate controls in their responses to mechanical stimulation, including noxious pin prick (Supplemental Figure 3A, http://links.lww.com/PR9/A324), or thermal stimuli (Supplemental Figure 3B, http://links.lww.com/PR9/A324).

### 3.3. Contactin-associated protein-like 2 overexpression reduces capsaicin-induced pain-like behaviours

**Figure 3.**
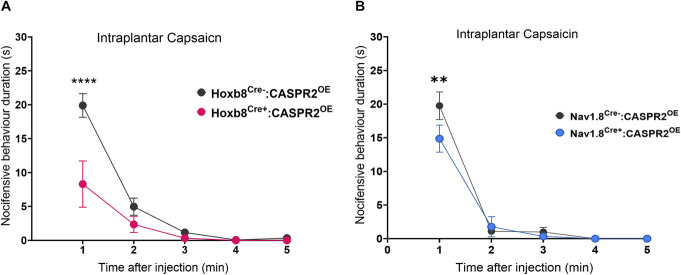
CASPR2 overexpression attenuates capsaicin-induced pain. (A) Hoxb8^Cre+^:CASPR2^OE^ mice (n = 5) exhibit significantly reduced nocifensive behaviour after intraplantar capsaicin injection (3 µg/paw) in the first minute when compared to littermate controls (n = 6). (B) Nav1.8^Cre+^:CASPR2^OE^ mice (n = 18) exhibit reduced nocifensive behaviour in the first minute after capsaicin injection, compared to littermate controls (n = 17). (A and B) Two-way RM ANOVA with Šídák multiple comparisons test, **P < 0.01, ****P < 0.0001. All data shown as mean ± SEM. CASPR2, contactin-associated protein-like 2.

Previous studies have shown that genetic ablation of CASPR2 in mice results in the marked hypersensitivity to capsaicin injection as measured by a significant increase in overt pain behaviours.^10,41^ The injection of capsaicin into the plantar skin of the hind paw rapidly elicited robust nocifensive responses that consisted of licking and shaking of the treated paw, which diminished over 5 minutes. Nocifensive responses to capsaicin were significantly reduced in Hoxb8^Cre+^:CASPR2^OE^ when compared to littermate controls (Fig. 3A). This was evident within the first minute after injection, the peak of pain behaviour (Fig. 3A). Interestingly, a significant reduction in nocifensive behaviour 1 minute after capsaicin injection was also observed in Nav1.8^Cre+^:CASPR2^OE^ mice (Fig. 3B). These findings demonstrate that the overexpression of CASPR2 in DRG neurons affects nociception, reducing responses to the noxious stimulant capsaicin, without affecting acute pain–related behaviour in response to mechanical or thermal stimulation.

### 3.4. Contactin-associated protein-like 2 expression is downregulated by nerve injury in the DRG

**Figure 4.**
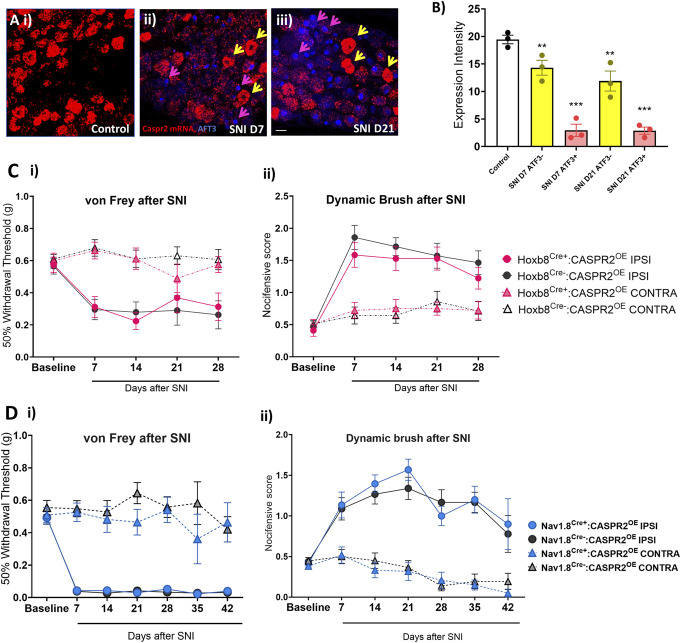
CASPR2 is downregulated in the DRG after nerve injury, but its overexpression does not affect pain-related hypersensitivity in the spared nerve injury (SNI) model. (A) Representative images of in situ hybridisation for CASPR2 mRNA expression (red) in WT mouse L4 DRG tissue sections (A-i) without injury or at (A-ii) 7 and (A-iii) 21 days after SNI. Atf3 immunoreactivity (blue) was used to mark injured neurons (Atf3+) as indicated by pink arrows, yellow arrows indicate noninjured (Atf3−) neurons. (B) Quantification of CASPR2 mRNA expression shows a significant downregulation at both 7 and 21 days after SNI. CASPR2 mRNA downregulation is most prominent in Atf3+ neurons (n = 3 animals per group). One-way ANOVA **P < 0.01, ***P < 0.001 vs control. Scale bar 25 µm. (C) SNI caused sustained mechanical hypersensitivity in the ipsilateral (IPSI) hind paw in all mice. (C–i) There was no difference in punctate mechanical hypersensitivity as measured by von Frey hairs, between Hoxb8^Cre+^:CASPR2^OE^ (n = 9) and Hoxb8^Cre−^:CASPR2^OE^ (n = 7) mice after nerve injury. (C-ii) There was also no difference in dynamic allodynia between the 2 genotypes after injury. Contralateral (CONTRA) sensitivity remained at baseline levels. (D) There was no difference in (D-i) punctate mechanical hypersensitivity, measured by von Frey hairs, between Nav1.8^Cre+^:CASPR2^OE^ (n = 15) and Nav1.8^Cre−^:CASPR2^OE^ (n = 17) mice after nerve injury. (D-ii) There was also no difference in dynamic allodynia between the 2 genotypes. Two-way RM ANOVA with Šídák multiple comparisons test. All data shown as mean ± SEM. CASPR2, contactin-associated protein-like 2.

Given that axotomised mouse DRG neurons in culture downregulate CASPR2 mRNA,^10^ we assessed whether peripheral nerve injury modified CASPR2 expression in vivo. Using in situ hybridisation (ISH),^10^ we found significant downregulation of CASPR2 in the L4 DRG of injured WT (wild type) mice using the spared nerve injury (SNI) model (Figs. 4A and B). This downregulation was evident at both 7 (Fig. 4A-ii) and 21 (Fig. 4A-iii) days after injury, time points selected to coincide with robust pain-related hypersensitivity. Using Aft3 (activating transcription factor 3) to mark injured neurons (Fig. 4A-ii, iii), the downregulation of CASPR2 is significant in Atf3 (uninjured) neurons, but greater in those expressing Atf3 at both time points, when compared to uninjured control (Fig. 4B).

### 3.5. Contactin-associated protein-like 2 overexpression does not affect neuropathic pain–related behaviours

In view of the downregulation of CASPR2 at time points correlated with nerve injury–induced pain-related behaviour in mice, next we assessed the impact of CASPR2 overexpression in the SNI model. We assessed changes to punctate and dynamic mechanical pain sensitivity using von Frey hairs (Fig. 4C-i) and dynamic brush (Fig. 4C-ii). Although there was a marked decrease in withdrawal threshold in the ipsilateral paw to Von Frey hair stimulation, no differences in response thresholds were observed between Hoxb8^Cre+^:CASPR2^OE^ mice and littermate controls (Fig. 4C-i). Increased nocifensive behaviour in response to dynamic brush was seen on the ipsilateral paw after nerve injury, indicative of the development of mechanical allodynia, which developed to similar levels in both genotypes (Fig. 4C-ii). No differences were seen in contralateral sensitivity after nerve injury between genotypes or when compared to baseline levels (Fig. 4C). Similarly, we found no difference in the development of mechanical pain–related hypersensitivity between Nav1.8^Cre+^:CASPR2^OE^ mice and littermate controls after nerve injury (Fig. 4D-i-ii). It is possible that CASPR2 overexpression is itself disrupted by nerve injury or that it does not recover expression back to preinjury levels given the downregulation of native CASPR2. However, using primers that detect both mouse and human transcripts, we find that CASPR2 mRNA remains overexpressed in injured DRG (Supplemental Figure 4, http://links.lww.com/PR9/A324), suggesting that, in these models, increasing the levels of CASPR2 is insufficient to attenuate evoked neuropathic pain behaviours.

### 3.6. The effects of contactin-associated protein-like 2 overexpression on Kv1 currents in DRG neurons

**Figure 5.**
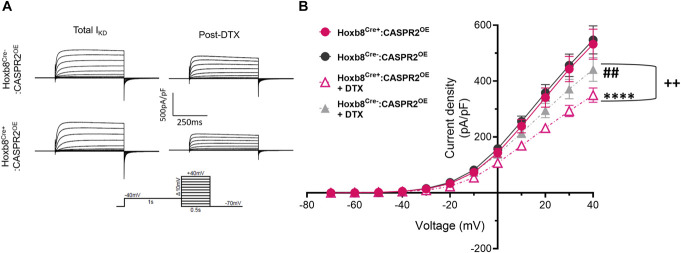
CASPR2 overexpression in Hoxb8^Cre+^:CASPR2^OE^ mice does not alter I_KD_ in DRG neurons but facilitates a larger contribution from α-DTX-sensitive Kv channels. (A) Example traces of outward current in medium sized DRG neurons (25–35 µm diameter) from both Hoxb8^Cre+^:CASPR2^OE^ and Hoxb8^Cre−^:CASPR2^OE^ mice evoked by depolarizing pulses in the before and after α-DTX application. (B) I_KD_ was measured before and after 100 nM of α-DTX. Current voltage relationships for I_KD_ show no difference between genotypes pre α-DTX. α-DTX treatment significantly reduced I_KD_ in DRG neurons from both Hoxb8^Cre−^:CASPR2^OE^ and Hoxb8^Cre+^:CASPR2^OE^ vs pretreatment. The reduction in I_KD_ caused by α-DTX was significantly greater in DRG neurons overexpressing CASPR2 (Hoxb8^Cre+^:CASPR2^OE^) vs control (Hoxb8^Cre−^:CASPR2^OE^). Cells taken from 3 mice per genotype (Hoxb8^Cre−^:CASPR2^OE^ n = 9 cells and Hoxb8^Cre+^:CASPR2^OE^ n = 11 cells). Two-way RM ANOVA, Sidak post hoc multiple comparisons test, ##*P* < 0.001 Hoxb8^Cre−^:CASPR2^OE^ + DTX vs Hoxb8^Cre−^:CASPR2^OE^; ****P < 0.0001 Hoxb8^Cre+^:CASPR2^OE^ + DTX vs Hoxb8^Cre+^:CASPR2^OE^, ++*P* < 0.01 Hoxb8^Cre−^:CASPR2^OE^ + DTX vs Hoxb8^Cre+^:CASPR2^OE^ + DTX. All data shown as mean ± SEM. α-DTX, α-dendrotoxin; CASPR2, contactin-associated protein-like 2.

Kv1 channels such as Kv1.1 and 1.2 are important molecular components of I_KD_, a slowly inactivating voltage-dependent potassium current active at subthreshold potentials, that is inhibited by α-DTX, a blocker of Kv1.1, 1.2, and 1.6.^19,26^ Previous work has shown that CASPR2 can regulate I_KD_ in DRG neurons.^10^ We, therefore, assessed the impact of CASPR2 overexpression on I_KD_ in DRG neurons from Hoxb8^Cre^:CASPR2^OE^ mice, focusing on medium diameter cells as done for genetic ablation studies.^10^ I_KD_ was measured before and after application of α-DTX (Fig. 5A). The current voltage relationships were determined and as expected α-DTX reduced I_KD_ in both genotypes (Fig. 5B, Supplemental Figure 5A-ii, iii, http://links.lww.com/PR9/A324). However, the reduction was greater in Hoxb8^Cre+^:CASPR2^OE^ neurons compared to controls (Fig. 5B, Supplemental Figure 5A-iv, http://links.lww.com/PR9/A324), suggesting a greater Kv1 contribution to I_KD_ in Hoxb8^Cre+^:CASPR2^OE^ neurons. Unexpectedly (and despite an increased α-DTX sensitive I_KD_), total I_KD_ was normal in Hoxb8^Cre+^:CASPR2^OE^ neurons compared to control (Fig 5B, Supplemental Figure 5A-i, http://links.lww.com/PR9/A324). These data suggest that CASPR2 overexpression does increase the membrane levels of α-DTX sensitive Kv1 subunits in DRG neurons, facilitating a greater contribution of these channels to I_KD_ than in control. However, this does not affect total I_KD_, proposing compensatory mechanisms that maintain I_KD_ at normal levels. The lack of effect on total I_KD_ may explain the limited action of CASPR2 overexpression on pain sensitivity.

## 4. Discussion

Antibodies against CASPR2 are associated with neuropathic pain in patients, and recent data have shown that the immune or genetic disruption of CASPR2 in mice results in the loss of Kv1 membrane expression, hyperexcitability in sensory neurons, and neuropathic pain-like behaviour.^10^ The downregulation of Kv1 channels in primary afferents after nerve injury is thought to contribute to neuropathic pain,^5,13^ and overexpression of CASPR2 in vitro reduces DRG neuron excitability.^10^ This led us to hypothesise that the overexpression of CASPR2 in vivo could reduce pain sensitivity and may represent a therapeutic option for the treatment of neuropathic pain. To test this theory, we generated a new transgenic mouse line and overexpressed CASPR2 selectively in either nociceptors or all DRG neurons.

Validation studies confirmed overexpression of CASPR2 in both Hoxb8^Cre+^:CASPR2^OE^ and Nav1.8^Cre+^:CASPR2^OE^ mice. Hoxb8^Cre+^:CASPR2^OE^ mice displayed robust overexpression of CASPR2 protein in both the sciatic nerve and DRG, with normal levels found in the brain, in line with the lack of Hoxb8 expression in this region.^39^ Nav1.8^Cre+^:CASPR2^OE^ mice also displayed significantly increased CASPR2 expression in the sciatic nerve at lower levels than Hoxb8^Cre+^:CASPR2^OE^ mice, with a trend towards increased CASPR2 at the level of the whole DRG. This likely reflects the smaller population of DRG neurons targeted (around 30% of DRG neurons are Nav1.8 negative). Contactin-associated protein-like 2 is a transmembrane protein exerting functional effects through regulating Kv1 channel expression at the cell membrane. Using cultured DRG neurons, we confirmed the overexpression of membrane CASPR2 in DRG neuron subpopulations using a CASPR2 antibody targeting an extracellular epitope. The binding intensity of this CASPR2 antibody to small diameter DRG neurons was comparable between both lines, suggesting that at the level of the cell a similar level of overexpression was induced. These findings validate our overexpression mouse line and highlight its utility for studying the role of CASPR2 in the nervous system.

We found that the overexpression of CASPR2 in all DRG neurons or nociceptors did not affect acute pain–related behaviours, as evidenced by the lack of significant difference in mechanical and thermal sensitivity between CASPR2-overexpressing and control mice. Studies in mice after Kv1.2 overexpression in DRG neurons also found a similar lack of alteration in acute pain sensitivity.^13^ Although our findings are in contrast to the increased mechanical and thermal pain–related sensitivity observed in CASPR2 KO mice,^10,41^ this may reflect a “ceiling effect” in the impact of increasing CASPR2 levels in relation to these modalities. However, we saw a significant reduction in nocifiensive behaviours in response to capsaicin paw injection. This effect was robust being observed in both Hoxb8^Cre+^:CASPR2^OE^ and Nav1.8^Cre+^:CASPR2^OE^ mice. This finding parallels with the hypersensitivity to capsaicin observed in CASPR2 KO mice and suggests that CASPR2 overexpression does affect nociception. Capsaicin specifically activates TRPV1 (the transient receptor potential vanilloid 1), a nonselective transduction channel expressed on nociceptors. A greater reduction in capsaicin-induced pain behaviour was observed in Hoxb8^Cre+^:CASPR2^OE^ when compared to Nav1.8^Cre+^:CASPR2^OE^ mice despite similar levels of overexpression in small diameter neurons between the 2 lines. Hoxb8 is also expressed in caudal spinal cord^39^; therefore, it is possible that spinal overexpression of CASPR2 may also have contributed to the reduction in capsaicin sensitivity.

We next wanted to evaluate the potential contribution of CASPR2 to nerve injury–induced pain. Using ISH in tissue from the SNI model, we found that traumatic nerve injury significantly reduced CASPR2 expression in DRG neurons at day 7 and 21 days after injury, time points associated with the development of mechanical pain-hypersensitivity.^11^ Using Atf3 as a marker, the downregulation of CASPR2 was more evident in injured neurons. Although both injured and uninjured afferents contribute to neuropathic pain, it has been suggested that injured fibres do so to a greater extent.^32,42^ Kv1 channels promote neuropathic pain through their downregulation, and these findings propose that the downregulation of CASPR2 may also contribute, limiting the membrane expression of an already diminished pool of Kv1 channels. However, we find that increasing the levels of CASPR2 in the DRG did not attenuate mechanical pain–related sensitivity in the SNI model in either overexpression line. One possibility for this lack of effect is that nerve injury reduces the levels of CASPR2 overexpression (as seen for native CASPR2). However, this does not seem to be the case since qPCR analysis of mRNA levels in ipsilateral nerve of Cre + mice showed that CASPR2 transcript levels remained elevated. Although some Kv1 subunits are upregulated after nerve injury (eg, Kv1.6),^5^ it is also possible that the effects of CASPR2 overexpression are restricted due to the downregulation of other subunits, such as Kv1.1 and 1.2, therefore, reducing the availability of CASPR2's functional substrates to levels insufficient to reverse hyperexcitability.

Since enhancing the membrane expression of Kv1 channels would be key in CASPR2 overexpression affecting pain sensitivity, we next measured Kv1 currents in DRG neurons to better understand the limited effects observed on pain sensitivity. We focused on I_KD_ a slowly inactivating outward current mediated by Kv1 channels.^15,26^ This current is sensitive to dendrotoxin (blocker of Kv1.1, 1.2, and 1.6) and significantly reduced in CASPR2 KO DRG neurons.^10^ When comparing DRG neurons from Hoxb8^Cre+^:CASPR2^OE^ mice with those from littermate controls, we found no differences in I_KD_. However, we did find that dendrotoxin application, which reduces I_KD_, did so to a greater extent in Hoxb8^Cre+^:CASPR2^OE^ neurons. These observations imply that CASPR2 overexpression is increasing the membrane trafficking of α-DTX-sensitive channels, resulting in their greater contribution to I_KD_, but it does this without altering total I_KD_. Kv1 channels are formed from tetra-heteromeric compositions of alpha subunits, with trafficking, function, and sensitivity to pharmacological agents dependent on the precise subunit configuration.^2,27,33^ It is, therefore, possible that the overexpression of CASPR2 may alter coassembly in a way that prevents an overall impact on I_KD_ or that increased trafficking triggers compensatory mechanisms given the homeostatic regulation of these currents within the nervous system.^9,38,43^ Although the exact reasons remain unclear, ultimately the lack of impact on total I_KD_ by CASPR2 overexpression likely explains its limited effects on pain sensitivity.

We did, however, see a significant reduction in behavioural responses to capsaicin. It is of note that the increased response of CASPR2 KO mice to capsaicin is particularly marked,^10^ potentially reflecting a more predominant effect of CASPR2 in TRPV1 expressing sensory neurons. Single cell sequencing studies confirm the coexpression of CASPR2 and Kv1 channels in TRPV1+ neurons, although expression is also seen in other DRG neuron subpopulations and frequently at higher levels.^44,46^ CASPR2 exerts its functional effects through protein interactions, and although these have been investigated in the CNS,^3,7^ they have not in the DRG. For example, such studies have revealed an interaction of CASPR2 with the type I IP3 receptor (IP3-R1) in the cerebellum,^3^ a receptor known to directly interact with TRPV1 in DRG neurons.^31^ Therefore, it is conceivable that a more direct modulation of TRPV1 by CASPR2 may explain our findings, by-passing the need to alter general excitability through Kv1 channel regulation.

In summary, CASPR2 overexpression does alter nociception, reducing sensitivity to the noxious stimulant capsaicin, but has limited effects on Kv1 mediated currents in DRG neurons and is insufficient to attenuate neuropathic pain in the SNI model. Although CASPR2 remains of interest in relation to the pathogenesis of neuropathic pain being one of the best validated autoantibody-mediated acquired pain disorders, the efficacy of therapeutics based on increasing CASPR2 levels (for instance using gene therapy) to the extent we have generated are likely to be limited due to compensatory mechanisms.

## Disclosures

S.R.I. has received honoraria/research support from Amgen, Argenx, UCB, Roche, Janssen, IQVIA, Clarivate, Slingshot Insights, Cerebral therapeutics, BioHaven therapeutics, CSL Behring, and ONO Pharma and receives licensed royalties on patent application WO/2010/046716 entitled “Neurological Autoimmune Disorders,” and has filed 2 other patents entitled “Diagnostic method and therapy” (WO2019211633 and US app 17/051,930; PCT application WO202189788A1) and “Biomarkers” (WO202189788A1, US App 18/279,624; PCT/GB2022/050614). D.B. has acted as a consultant for 5 AM ventures, AditumBio, Astra Zeneca, Biogen, Biointervene, Combigene, GSK, Lexicon therapeutics, Neuvati, Novo Ventures, Olipass, Orion, Replay, Replay, SC Health Managers, Third Rock ventures, Vida Ventures, Vertex on behalf of Oxford University Innovation.

## Appendix A. Supplemental digital content

Supplemental digital content associated with this article can be found online at http://links.lww.com/PR9/A324.

## Supplementary Material

SUPPLEMENTARY MATERIAL
