# Supplementary material for: Specific overexpression of contactin-associated protein-like 2 and its effects on pain-related behaviour in mice
Source: Pain Rep. 2025 Jun 26;10(4):e1309. doi: 10.1097/PR9.0000000000001309 (PMC12204150; doi:10.1097/PR9.0000000000001309)
Supplement: SUPPLEMENTARY MATERIAL [file painreports-10-e1309-s001.pdf]

Supplementary materials

Supplementary Figure 1

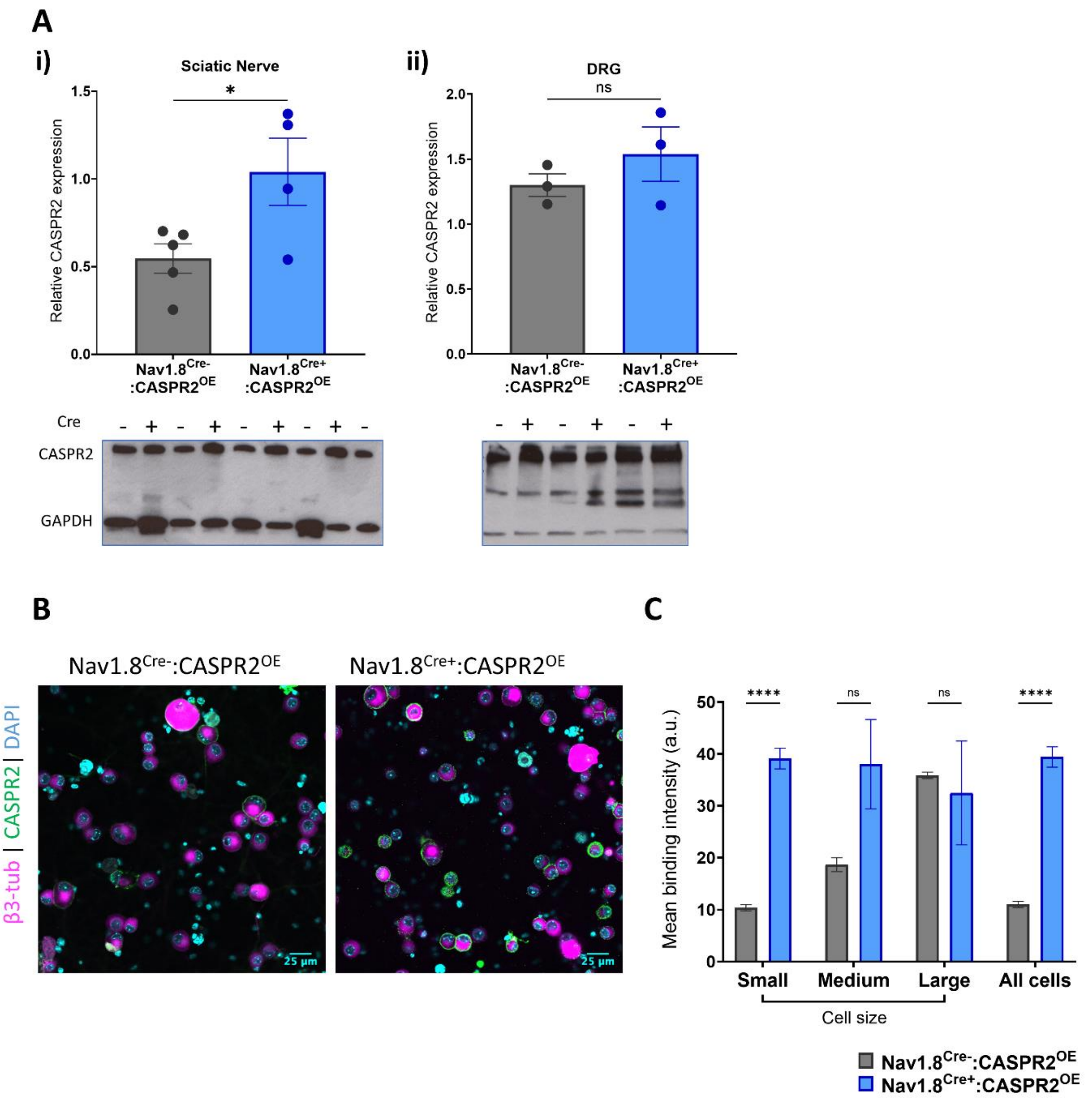

Supplementary Figure 1. CASPR2 overexpression in Nav1.8^{Cre+}:CASPR2^{OE} mice. A) Quantification of CASPR2 protein in the **A-i)** sciatic nerve and **A-ii)** DRG. **A-i)** CASPR2 protein is statistically significantly overexpressed in the sciatic nerve of Nav1.8^{Cre+} mice. **A-ii)** There is a trend to increased CASPR2 expression in the DRG, but this did not reach statistical significance. Two-tailed unpaired t test, * $p<0.05$, n=3-4. Images of immunoblots shown below. **B)** Representative images of CASPR2 expression (green) on the soma of Nav1.8^{Cre-}

:CASPR2^{OE} (left) and Nav1.8^{Cre+}:CASPR2^{OE} (right) DRG neurons (cultured for 2 days in vitro) DAPI (cyan) and βIII-tubulin (magenta). Scale bar 25μm. **C)**. Quantification of CASPR2 expression on the surface of DRG neurons, separated by cell size; small (<25μm in diameter), medium (25-35μm), and large (>35μm). CASPR2 stained with an antibody targeting an extracellular epitope on live neurons. DRG neurons pooled from 2 animals/genotype. CASPR2 is significantly overexpressed on the surface of small-sized neurons in Nav1.8^{Cre+}:CASPR2^{OE} compared to controls. Nav1.8^{Cre+}:CASPR2^{OE} (201 cells), Nav1.8^{Cre-}:CASPR2^{OE} (276 cells). Two-way ANOVA with Šídák's multiple comparisons test, ****p<0.0001, n=cells. All data shown at mean±SEM.

Supplementary Figure 2

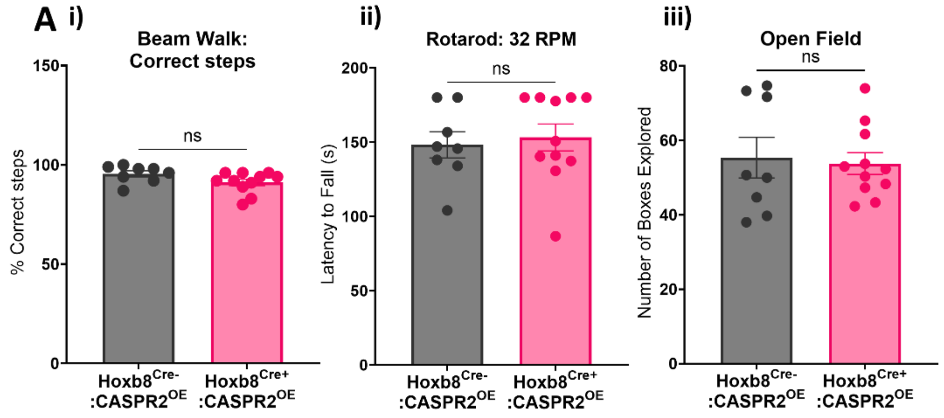

Supplementary Figure 2. Hoxb8^{Cre+}:CASPR2^{OE} mice display normal proprioception and motor activity. A) When compared to littermate controls, Hoxb8^{Cre+}:CASPR2^{OE} mice have a similar performance on the **A-i)** beam test, as measured by the percentage of correct steps, **A-ii)** the Rotarod, as measured by the latency to fall when set at a speed of 32rpm and **A-iii)** the open field, as measured by the number of boxes entered within a 3 minute period. Two-tailed unpaired t-test. Hoxb8^{Cre-}:CASPR2^{OE} *n*=8; Hoxb8^{Cre+}:CASPR2^{OE} n=11. All data shown as mean±SEM.

Supplementary Figure 3

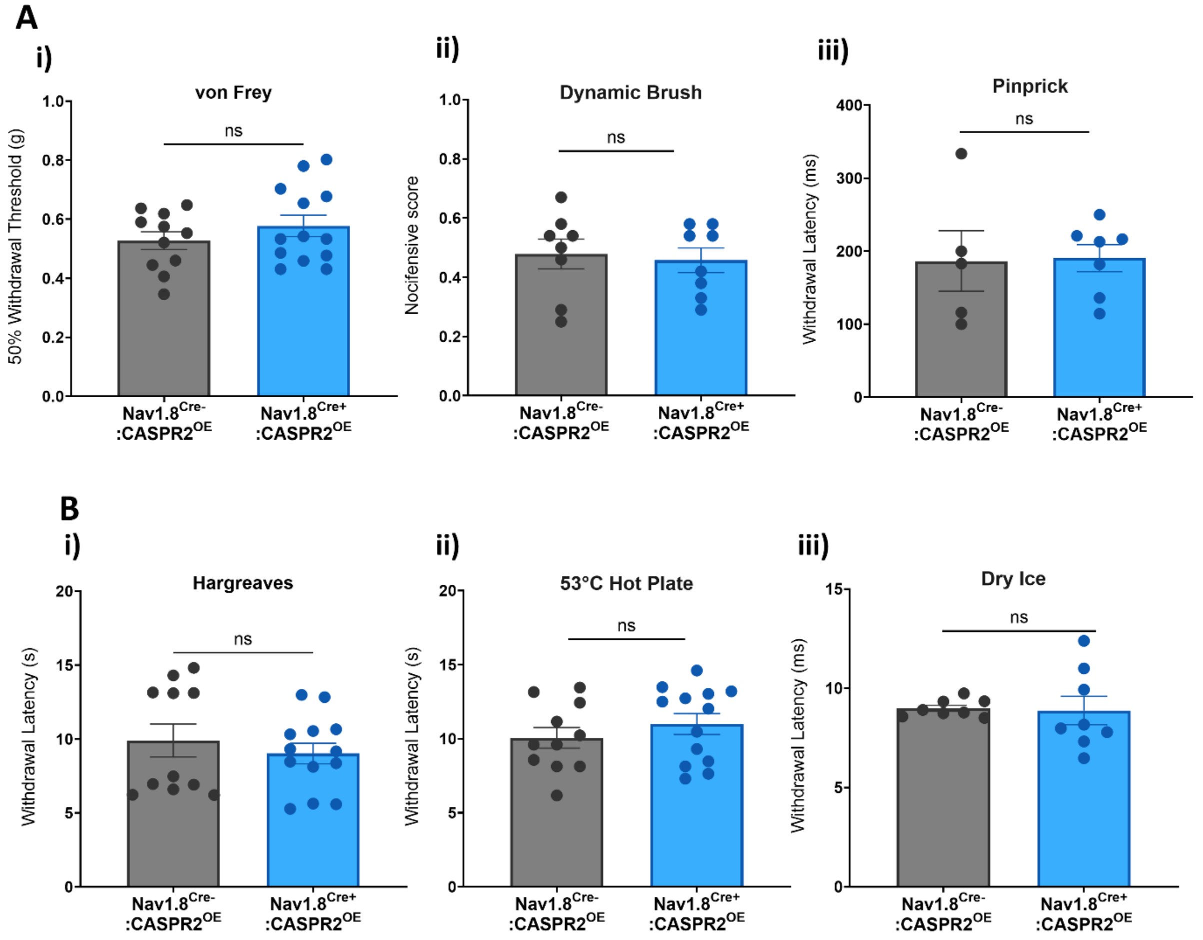

Supplementary Figure 3. CASPR2 overexpression in Nav1.8^{Cre+}:CASPR2^{OE} mice does not impact acute pain-related behavior. A) There were no significant differences in mechanical sensitivity detected between Nav1.8^{Cre+}:CASPR2^{OE} (n=7-13) and Nav1.8^{Cre-}:CASPR2^{OE} control littermates (n=5-11) as measured by **A-i)** von Frey hairs, **A-ii)** dynamic brush, or **A-iii)** pinprick test. Two-tailed unpaired t-test. **B)** There were no significant differences in thermal sensitivity detected between genotypes as measured by **B-i)** Hargreaves test **B-ii)** 53°C hot plate, or **B-iii)** dry ice assay. Two-tailed unpaired t-test. All data shown as mean±SEM.

Supplementary Figure 4

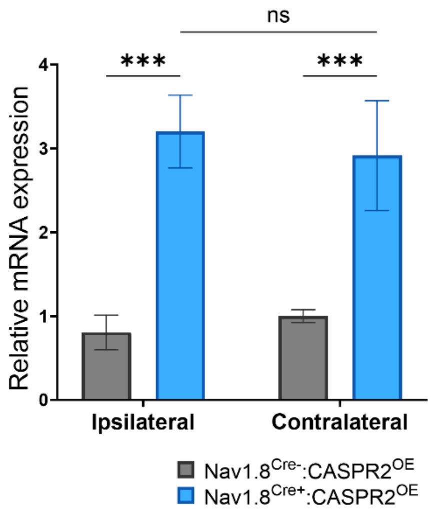

Supplementary Figure 4. CASPR2 overexpression is maintained after nerve injury. Using qPCR to measure mRNA levels in ipsilateral and contralateral DRG (L3-5), 28 days after SNI. The overexpression of Caspr2 was sustained in the Nav1.8^{Cre+}:CASPR2^{OE} mice after injury, n=4. Two-way RM ANOVA with Šídák's multiple comparisons test, ***p<0.001. All data expressed at mean±SEM.

Supplementary Figure 5

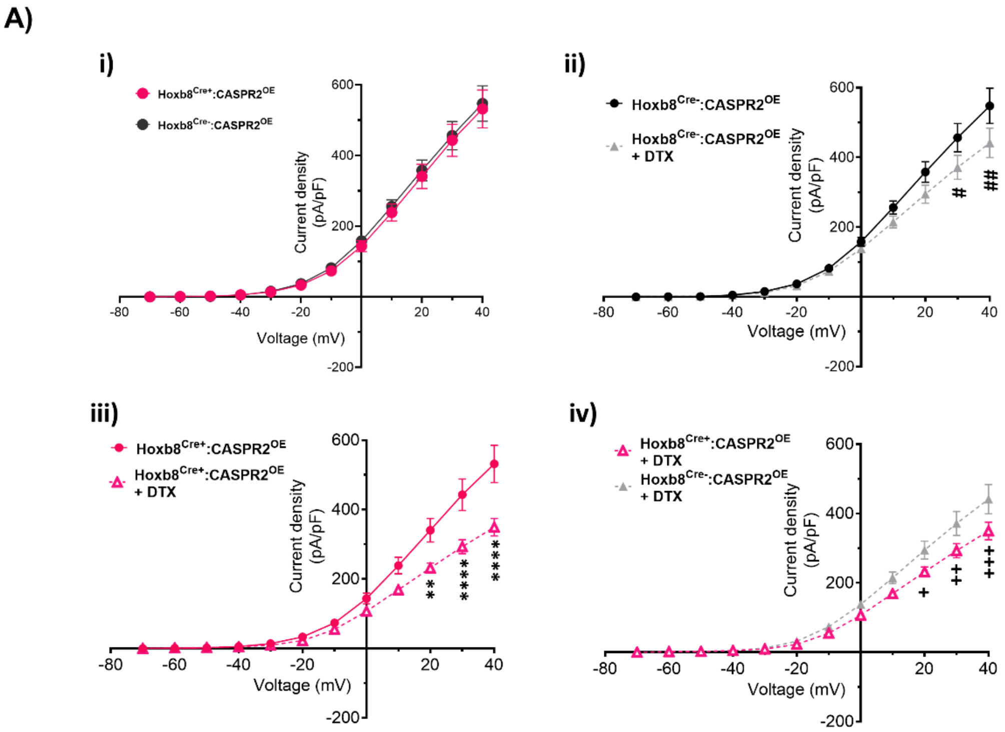

Supplementary Figure 5. CASPR2 overexpression in Hoxb8^{Cre+}:CASPR2^{OE} mice does not alter I_{KD} in DRG neurons, but facilitates a larger contribution from α-DTX-sensitive Kv channels. A-i) No difference in total I_{KD} between genotypes. **A-ii,iii)** α-DTX significantly reduces I_{KD} in both Hoxb8^{Cre-}:CASPR2^{OE} **(A-ii)** and Hoxb8^{Cre+}:CASPR2^{OE} **(A-iii)** neurons versus controls. A-iv) the reduction in I_{KD} is significantly greater in DRG neurons from Hoxb8^{Cre+}:CASPR2^{OE} versus Hoxb8^{Cre-}:CASPR2^{OE} mice following α-DTX treatment. (Hoxb8^{Cre-}:CASPR2^{OE} n=9 cells and Hoxb8^{Cre+}:CASPR2^{OE} n=11 cells). Cells taken from 3 mice per genotype. Two-way RM ANOVA, Sidak's post-hoc multiple comparisons test, #$p<0.05$, ##$p<0.01$, **$p<0.01$, ***$p<0.001$, ****$p<0.0001$, +$p<0.05$, ++$p<0.01$, +++$p<0.001$. All data shown as mean±SEM.

Supplementary Table 1:

Gene	Forward primer	Reverse primer
CNTNAP2	GACAGATGTGTGCCCAATCAC	GTGCATTTGAAGCTGTCCCA
18s	GGACCAGAGCGAAAGCATTTG	GCCAGTCGGCATCGTTTATG
Hprt1	GTCCTGTGGCCATCTGCCTAG	TGGGGACGCAGCAACTGACA
Gapdh	TGTGTCCGTCGTGGATCTGA	TTGCTGTTGAAGTCGCAGGAG

Table S1. Sequences for primer pairs used for QPCR

Supplementary methods:

Behavioural testing

Pinprick: Pinprick test assesses the response to noxious mechanical stimulus. Mice were randomly assigned and acclimatized to a test box (5x5x10cm) elevated on a wire mesh base. Mice were then tested on their plantar hind paws using a dissection pin attached to a 1g calibrated von Frey filament [1] The latency to withdrawal was recorded using an iPhone XS (Apple) at 240 fps (4.14 ms / frame) and analysed using Avidemux 2.7.2. Responses from left and right paws were averaged on three different days.

Beam walk: The apparatus is a round wooden beam of about 1 m in length and 2 cm in diameter elevated from the bench surface (made in-house). Mice were briefly acclimatised to the wooden beam apparatus before testing. The time of crossing and the percentage of correct steps was recorded; The percentage of missteps was calculated by counting the number of missed steps as a percentage of total steps taken to cross the beam. An average was taken from three testing days.

Rotarod: Mice were acclimatised and briefly trained on the Rotarod apparatus (Ugo Basile) until all mice were able to complete a minimum of 30 seconds at 28 RPM. The latency to fall on the 32 RPM was recorded on three separate days and averaged. The maximum testing time for all mice is 180 seconds.

Open field: Mice were not acclimatised to the open field apparatus as this is a novelty and exploratory test. Mice were placed in a black box with a grid system on the box floor, and the number of boxes the mouse entered during a three-minute period was recorded. An average was obtained from two tests.

Spared nerve injury

Mice were anaesthetised using 2% isoflurane and prepared for surgery by shaving and sterilising the thigh. A small incision was made to the thigh skin parallel to the thigh bone. The sciatic nerve was identified through separation of muscle and connective tissues. The point where the sciatic nerve trifurcates was identified, and sutures were tied around the nerves for transection. Non-absorbable sutures were used for the nerve ligation. For the spared sural nerve injury model, the common peroneal and tibial nerves were ligated, and the nerves were transected while removing ~1 mm of the transected nerve (this model was used for $Na_v1.8^{Cre}$:$CASPR2^{OE}$ behaviour and for ISH analysis of CASPR2 expression) . For the spared tibial nerve injury model, the sural and common peroneal nerves were ligated separately and transected while removing ~1 mm of the transected nerves to prevent regeneration (this model was used for $Hoxb8^{Cre}$:$CASPR2^{OE}$ behaviour). The incision site was closed with an absorbable suture and the skin was closed with surgical staples. Appropriate post-operative pain medications were given with a local injection of 2 mg/kg Marcain (AstraZeneca) and a systemic injection of 5 mg/kg Rimadyl (Pfizer). Mice were monitored daily for changes to wound healing, autotomy, and body weight.

Western Blot

DRGs and sciatic nerves (~1cm) were isolated and homogenised on ice in RIPA buffer (Sigma) with protease inhibitors (Roche). Insoluble cell debris was removed by centrifugation (12,000 RPM, 15 min). Protein concentration of the extract was quantified using the BCA assay (Pierce). Extracts were separated by SDS-PAGE (4-20% gel, Bio-Rad) then transferred onto nitrocellulose membranes. Membranes were blocked with PBS-milk and incubated overnight with primary antibodies: anti-CASPR2 antibody (1:200 dilution, Abcam) and an anti-GAPDH antibody (1:5,000 dilution, Abcam) as a loading control. The blots were washed with PBS-Tween then probed with HRP-labelled secondary antibodies and processed for chemiluminescence detection (ECL, Amersham). For fluorescent detection, Alexa 488, Alexa 546, or Dylight 680-conjugated secondary antibodies were used at a 1:3,000 dilution. Immunoblots were imaged using the ChemiDoc imager (Bio-rad). Analysis was performed using the Image Studio software (Li-Cor).

Quantitative Real Time PCR (qPCR)

RNA was isolated from fresh-frozen mouse DRGs. RNA was isolated using a combination of TriPure (Roche) and a High Pure RNA tissue kit (Roche). Briefly, tissue was homogenised in

Tripure using a handheld homogeniser (Cole-Parmer), treated with chloroform and then column-purified and eluted in RNase-free water. cDNA synthesis was carried out using Transcriptor reverse transcriptase (Roche), random hexamers (Invitrogen) and dNTPs (Roche).

Gene expression was quantified by detecting amplified material using LightCycler SYBR Green Master Mix (Roche) on the LightCycler 480 II system (Roche). Three technical replicates were included for each sample. Primer pair against CNTNAP2 recognised both mouse and human transcripts. Results were normalized to three reference genes controls (18s, GAPDH and HPRT1) using the $\Delta\Delta C_T$ method. Primer sequences shown in Supplementary Table 1.

Primary DRG neuron culture:

Adult mice were sacrificed in a CO_2 chamber. The spinal column was removed and bisected, and DRGs were taken at all levels and placed into Hanks' Balanced Salt Solution (HBSS, without Ca^{2+} and Mg^{2+}, Invitrogen). DRG were digested enzymatically at 37°C for 1.5 hrs in Collagenase II (4 mg/mL, Gibco) and Dispase II (4.7 mg/mL, Roche) diluted in HBSS. DRG were mechanically dissociated by gentle trituration with a fire-polished glass pipette and washed in HBSS. Dissociated cells were suspended in culture medium (Neurobasal medium with 2% B27 (v/v) and 1% glutaMAX (v/v), Gibco) supplemented with mouse NGF (50 ng/µl, Peprotech) and GDNF (10 ng/µL, Peprotech) and plated in laminin / poly-D Lysine-coated 96-well plates (Greiner) or coverslips. Cultured cells were incubated at 37°C with 5% CO_2 for two days until assay.

Live cell staining

Cultured DRG neurons were treated with a human monoclonal antibody targeting the extracellular domain of CASPR2 (generated in house) [2] at a concentration of 20µg/ml and incubated for one hour at 37°C. The subsequent steps were all completed at room temperature. The medium was removed, and cells were washed twice and then incubated with Alexa 488-goat anti-human IgG (H+L) for an hour. The cells were washed twice, then fixed with 4% PFA (paraformaldehyde) and permeabilised with 0.3% Triton-X diluted in PBS. The neuronal marker, Alexa 546-rabbit anti-βIII tubulin antibody (Abcam), was added and incubated with cells for one hour. After an hour of incubation, the cells were washed and mounted on slides with Vectashield mounting medium (Vector Labs). Slides were imaged using the Zeiss LSM 700 using 405nm, 488nm, and 546nm diode lasers. Imaging was

performed at the z-plane needed to capture cell bodies for analysis. Images were processed using FIJI (ImageJ).

Analysis of antibody binding on cell membrane

Analysis of IgG binding to muDRG neurons was performed using FIJI (Image J). Briefly, the cell membranes were identified by thresholding or manually delineating the tubulin marker. The cells or neurons were then added as regions of interest (ROIs). The ‘Enlarge’ and ‘Make band’ tools were used to create a band surrounding the membrane and the mean intensities of the identified bands were measured. To facilitate the analysis of large numbers of images, an ImageJ macro (unpublished) was written. The mean intensity data was exported to Excel or GraphPad Prism9 for further analysis.

In situ hybridisation (ISH) and immunohistochemistry (IHC)

Control (uninjured) or mice 7 and 21 days after SNI surgery were SNI 7 and 21 days after surgery were overdosed with pentobarbital and transcardially perfused with saline and then 4% PFA (paraformaldehyde). L4 DRG were removed, post-fixed in 4% PFA for 1.5 hours and cryoprotected in 30% sucrose at 4°C for 24 hours. Individual DRG were embedded in OCT and stored at -80 before sectioning on to superfrost plus slides at a thickness of 10µm. ISH was performed using the RNAScope 2.5 RED chromogenic assay kit and a hybridisation oven, following the manufacturer’s protocol (Advanced Cell Diagnostics). Briefly, tissue sections were washed in PBS and pre-treated with hydrogen peroxide at room temperature, followed by a protease treatment at 40°C for 10minutes. Slides were incubated with a 20zz probe to detect CASPR2 mRNA targeting position 3708-5086 of NM_001004357.2 for 2 hours. Slides were then subjected to 6 rounds of amplification followed by detection with fast red. Slides were then washed with PBS-Tx (0.3%) and incubated with a primary rabbit antibody against ATF3 (Santa Cruz) at 1:500 overnight at room temperature. Slides were washed and then incubated with an anti-rabbit secondary antibody. Sections were then immersed in mounting media, coverslipped and images acquired using a confocal microscope (Zeiss) and the Zen black software. Z-stacks were taken and maximum projection images created. Image analysis was performed using ImageJ. Neurons were manually circled. The intensity of red signal calculated for each cell profile and an average taken for each mouse (>100 cells from 3-4 sections). ATF3 positivity was defined by eye.

Statistical analysis

Student's t-test was used to compare the mean of two groups. In experimental groups where multiple comparisons were made, one-way or two-way analysis of variance (ANOVA) tests with appropriate post-hoc tests were performed. Fisher exact test was used to compare discrete binary variables. All data is represented as mean ± the standard error of the mean (SEM) unless otherwise stated. Statistical significance for all experiments was placed at $p < 0.05$. Statistical significance is indicated as follows * $p < 0.05$, ** $p < 0.01$, *** $p < 0.001$, **** $p < 0.0001$. The statistical tests used are reported in the appropriate figure legend. All statistical tests were carried out with GraphPad Prism 9.

References:

[1] Arcourt A, Gorham L, Dhandapani R, Prato V, Taberner FJ, Wende H, Gangadharan V, Birchmeier C, Heppenstall PA, Lechner SG. Touch Receptor-Derived Sensory Information Alleviates Acute Pain Signaling and Fine-Tunes Nociceptive Reflex Coordination. Neuron 2017;93:179–193. doi:10.1016/j.neuron.2016.11.027.

[2] Sun B, Fernandes D, Kienzler A-K, Paneva S, Harrison R, Ramanathan S, Harrison AL, Makuch M, Fichtner ML, Donat RF, Akdeniz D, Bayuangga H, Im MG, Williams R, Vasconcelos A, Thomsen S, Fower A, Sun R, Fox H, Mgbachi V, Davies A, Tseng M, Handel A, Kelly M, Zhao M, Bancroft J, Bashford-Rogers R, Pluvinage J V, Dandekar R, Alvarenga BD, Dustin L, Rinaldi S, Owens R, Anthony D, Bennett DL, Waters P, Davis SJ, Wilson MR, O'Connor KC, Soltys J, Carvalho AL, Irani SR. Permissive central tolerance plus defective peripheral checkpoints licence pathogenic memory B cells in CASPR2-antibody encephalitis. bioRxiv 2025. doi:10.1101/2025.01.14.631703.
